# Metal-Macrofauna Interactions Determine Microbial Community Structure and Function in Copper Contaminated Sediments

**DOI:** 10.1371/journal.pone.0064940

**Published:** 2013-05-31

**Authors:** Daniel J. Mayor, Nia B. Gray, Joanna Elver-Evans, Andrew J. Midwood, Barry Thornton

**Affiliations:** 1 Institute of Biological and Environmental Sciences, Oceanlab, University of Aberdeen, Aberdeen, United Kingdom; 2 The James Hutton Institute, Craigiebuckler, Aberdeen, United Kingdom; 3 School of Chemistry, University of Edinburgh, Edinburgh, United Kingdom; Royal Netherlands Institute of Sea Research (NIOZ), The Netherlands

## Abstract

Copper is essential for healthy cellular functioning, but this heavy metal quickly becomes toxic when supply exceeds demand. Marine sediments receive widespread and increasing levels of copper contamination from antifouling paints owing to the 2008 global ban of organotin-based products. The toxicity of copper will increase in the coming years as seawater pH decreases and temperature increases. We used a factorial mesocosm experiment to investigate how increasing sediment copper concentrations and the presence of a cosmopolitan bioturbating amphipod, *Corophium volutator*, affected a range of ecosystem functions in a soft sediment microbial community. The effects of copper on benthic nutrient release, bacterial biomass, microbial community structure and the isotopic composition of individual microbial membrane [phospholipid] fatty acids (PLFAs) all differed in the presence of *C. volutator*. Our data consistently demonstrate that copper contamination of global waterways will have pervasive effects on the metabolic functioning of benthic communities that cannot be predicted from copper concentrations alone; impacts will depend upon the resident macrofauna and their capacity for bioturbation. This finding poses a major challenge for those attempting to manage the impacts of copper contamination on ecosystem services, e.g. carbon and nutrient cycling, across different habitats. Our work also highlights the paucity of information on the processes that result in isotopic fractionation in natural marine microbial communities. We conclude that the assimilative capacity of benthic microbes will become progressively impaired as copper concentrations increase. These effects will, to an extent, be mitigated by the presence of bioturbating animals and possibly other processes that increase the influx of oxygenated seawater into the sediments. Our findings support the move towards an ecosystem approach for environmental management.

## Introduction

Trace levels of copper are essential for the healthy functioning of organisms owing to its central role in a range of enzymes [1]. However, this heavy metal is well known for its toxicity and the biocidal properties of copper have been exploited by mankind for centuries. Copper in the form of Cu_2_O is now the dominant active ingredient found in antifouling paints applied to marine vessels and other permanently submerged structures such as fish farm cages [2], [3] due to the global ban of organotin-based compounds in 2008. Cu^2+^ ions slowly leach from the paint and particulate copper is further released to the environment in flakes of paint produced during the periodic cleaning and maintenance of antifoulant-coated structures [2], [4], [5]. Moving ships are estimated to leach ∼100 tonnes of copper into the Greater North Sea each year, a value that does not include the considerable losses occurring in harbours and marinas [6]. Copper ions have a strong affinity for binding with particulate matter [7], which ultimately carries this heavy metal to the seafloor. Relatively low biological demands for copper and the typically reducing nature of marine sediments result in the accumulation of this element at the seabed [8], with concentrations in ship recycling zones, beneath fish farms and near boat yards reaching up to 703, 805 and 2230 mg Cu [kg dry sediment]^−1^ respectively [9], [10], [11].

The global growth in demand for sea freight, which increased from 28,723 to 40,891 billion ton-miles between 2000 and 2010 [12], is causing concentrations of copper entering the marine environment to rise. Marine aquaculture activities are also increasing rapidly and represent another growing input of copper into coastal benthic ecosystems, through the use of copper-enriched feeds and antifouling products [9], [13]. Increased inputs of copper to marine ecosystems are occurring in concert with ocean warming and acidification. These processes are both expected to increase the bioavailability and hence toxicity of copper, potentially by ≥100% over the next 100 years [14], [15].

Copper is known to affect the biomass and metabolic activities of sediment-associated bacteria [3], [16], driving changes in their community structure [17], [18]. It also affects the activity and survival of marine metazoan fauna [19], impacting upon their capacity for bioturbation [20]. Bioturbation is the process by which faunal movements increase the flow of oxygenated water into the seabed, and is well known to influence sediment microbial community structure and nutrient effluxes [21], [22], [23]. Macrofaunal movements also increase the transport of heavy metals into sediments [24] and mobilize copper owing to the enhanced supply of oxidizing solutes [8] and organic copper-complexing compounds [25]. Previous mesocosm studies investigating the effects of copper on marine benthic communities have reported direct effects [26] and interactions with trophic complexity and nutrient availability [27], [28], [29]. An equally complex variety of responses to copper contamination are reported in multi-species aquatic microcosm experiments [30]. We hypothesized that copper contamination and bioturbation interactively affect the composition and metabolic functioning of soft sediment microbial communities. Our factorial mesocosm experiment focussed on the cosmopolitan bioturbating amphipod *Corophium volutator* because a) its ventilatory- and sediment reworking activities can significantly affect carbon and nitrogen cycling in marine sediments [31], [32] and b) long-term exposure to even low concentrations of copper is expected to negatively affect their population density [33]. We employed a combination of phospholipid fatty acid (PLFA) analyses and compound-specific isotope ratio mass spectrometry (IRMS) to examine the microbial response in our experiments. This combination of techniques enables the relative structure and metabolic functioning of extant microbial groups to be examined. The δ^13^C signature of individual fatty acids can provide information on the balance between catabolism and anabolism of particular PLFAs, carbon isotope fractionation, and also the identity of substrates used for biosynthesis [34], [35], [36].

## Materials and Methods

### Study Location and Sediment Preparation

Experimental animals and sediments were collected at low tide from the mudflats in the lower reach of the Ythan Estuary, Aberdeenshire, NE Scotland, UK (57° 20.085′N, 02° 0.206′W) on 12/10/2009. All necessary permissions for work on the Ythan and Forvie National Nature Reserve were obtained from Scottish Natural Heritage. No protected or endangered species were involved in our experiments. The marine amphipod, *C. volutator*, was removed from the upper 3 cm of sediment by gentle sieving (1 mm) and acclimated to laboratory conditions in fresh, aerated seawater for 24 hrs prior to experimentation. Bulk sediments from the upper 3 cm were gathered by hand and subsequently sieved (0.5 mm) to remove macrofauna and large organic debris. The resulting homogenised sediments contained 1.37±0.01% w/w organic carbon, δ^13^C −22.28±0.04 ‰ where error is ±1se and n = 5. Copper concentrations in these sediments range between 1.9–4.5 mg Cu [kg dry sediment]^−1^ [37]. Copper treatment levels (Tables 1 and S1), chosen to span the range of concentrations present in the natural environment [9], [10], [11], were established by thoroughly homogenizing a saline solution of copper (II) sulphate pentahydrate into known quantities of pre-sieved sediment [19]. This was chosen because the majority of antifouling paints use Cu_2_O and the main biocidal species obtained from this compound in the presence of O_2_ is Cu^2+^ [2]. All of the seawater used was pumped from the estuary at high tide (33 ppt), UV-sterilized and 10 µm filtered prior to use.

**Table 1:** Nominal copper concentrations and the number of animals surviving (± stdev).

Nominal copper concentration(mg Cu [kg wet sediment]^−1^)	Number of animals survivingafter 10 days of incubation
0.0 (0)	22.4±3.8
30.2 (50)	26.2±1.3
90.5 (150)	14.8±4.3
181.0 (300)	11.2±6.0
301.7 (500)	12.4±1.5
603.4 (1000)	5.4±2.9

Values in parentheses represent dry weight concentrations.

### Mesocosm Experiments

A total of 60 mesocosms were assembled to examine how increasing concentrations of copper and the presence of *C. volutator* affected nutrient release from the sediments and benthic microbial community structure. Individual mesocosms consisted of clear cylindrical cores (300 mm high, 100 mm internal diameter) fitted with removable acetyl baseplates. An 8 cm thick layer of sieved sediment at the required copper concentration was carefully introduced into each core and subsequently submerged beneath a 20 cm column (∼1.5 L) of UV-sterilized and 10 µm filtered seawater. Thirty healthy *C. volutator* (≥4 mm body length) were introduced to replicate (n = 5) mesocosms at each treatment level. This is equivalent to 3820 individuals m^−2^, a density chosen to be equivalent to or lower than that found at the sampling location. *C. volutator* are deposit- and episammic feeders in the absence of suspended particulate matter [38]. The organic carbon content of the sediments was greatly in excess of the respiratory demands of these animals over the experimental duration (Text S1). The remaining mesocosms at each concentration of copper (n = 5) were incubated without *C. volutator*. All experimental units were incubated at 15°C with a 12 h light:dark cycle and were continuously aerated throughout the 10 day experimental period to ensure that the water was saturated with oxygen. Cores were examined daily and any dead animals on the sediment surface were removed via a glass tube. However, in many cases dead animals were lost from the experiment through decomposition beneath the sediment surface. Water samples to determine nutrient concentrations were collected at the end of the experiment. The remaining overlying water was then carefully removed from each core and sediment samples from the upper 1 cm were collected for subsequent analysis of the phospholipid fatty acid (PLFA) content. All nutrient and PLFA samples were stored frozen (−20°C) prior to analysis. Surviving animals were retrieved by sieving the experimental sediments.

### Analytical Techniques

Concentrations of dissolved NH_4_^+^-N, NO_x_^–^N and PO_4_^3–^P (collectively ‘nutrients’ hereafter) were determined with a modular flow injection auto-analyser (FIA Star 5010 series) using an artificial seawater carrier solution. Sediment organic carbon isotopic composition was determined on pre-acidified samples using a Flash EA 1112 Series Elemental Analyser connected via a Conflo III to a Delta^Plus^ XP IRMS (Thermo Finnigan, Bremen, Germany). Purified PLFAs were extracted from freeze-dried sediment samples and derivitized to yield fatty acid methyl esters (FAMEs) [39], [40]. The concentrations and carbon isotope ratios of individual FAMEs were measured using a GC Trace Ultra with combustion column attached via a GC Combustion III to a Delta V Advantage IRMS (all Thermo Electron, Bremen, Germany). Individual PLFAs were quantified by combining the area of their mass peaks, m/z = 44, 45 & 46, after background subtraction, and comparison with a known internal standard (19∶0) added to each sample [41]. Bacterial biomass was calculated from concentrations of the biomarker PLFAs i15∶0, ai15∶0 and i16∶0, assuming these represent 10% of total bacterial PLFAs and 0.056 gC PLFA/gC biomass [42]. Carbon isotope ratios of individual PLFAs were calculated with respect to Vienna-PDB (δ^13^C_V-PDB_) through the use of a CO_2_ reference gas injected before every sample and traceable to International Atomic Energy Agency reference material NBS 19 TS-Limestone. Repeated analysis over a two month period of the δ^13^C value of a C19 FAME internal standard gave a standard error of 0.26 ‰ (n = 18). PLFA-derived data relates only to the extant microbial community as PLFAs in non-living biomass undergo rapid environmental degradation.

### Data Analysis

Data exploration was undertaken to identify outliers and instances of collinearity [43]. The effect of copper concentration on the survival of *C. volutator* was examined using linear regression. Proportional survival data are bounded by 0 and 1 and were therefore square root arcsin transformed prior to analysis. The median concentration of copper that caused 50% mortality of *C. volutator* was estimated using the trimmed Spearman-Karber method using software supplied by the U.S. Environmental Protection Agency [44]. Partial linear regression analysis of each nutrient dataset was undertaken to examine the relative importance of copper concentration and the transformed proportion of surviving *C. volutator*. In all 3 models, ≥38.4% of the explained variance was attributable to copper concentration and ≤1.1% was solely attributable to *C. volutator* (Table S2). Subsequent analysis of the three nutrient datasets using generalized least squares (GLS) regression included copper concentration as a nominal variable to allow for non-linear effects and *C. volutator* as a binary variable (present/absent) to avoid collinearity issues with copper level. Bacterial biomass data were analysed similarly. All GLS regression models were subjected to a hierarchical backwards selection procedure using likelihood ratio (L. Ratio) tests to remove non-significant terms. Full details of this procedure and subsequent model validation are presented elsewhere [13], [45], [46]. Redundancy analysis (RDA) was used to investigate how the proportional abundance and delta values of individual PLFAs were influenced by nominal copper concentration, the presence/absence of *C. volutator* and the interaction between these variables. The significance of individual model terms was determined using a permuted (n = 9999) forwards selection procedure analogous to that employed in the CANOCO software [47]. All statistical analyses were conducted in the ‘R v2.11.1’ programming environment [48] using the ‘nlme’ [49] and ‘vegan’ [50] packages.

## Results

### Copper Effects on Survival of *C. volutator*

**Figure 1:**
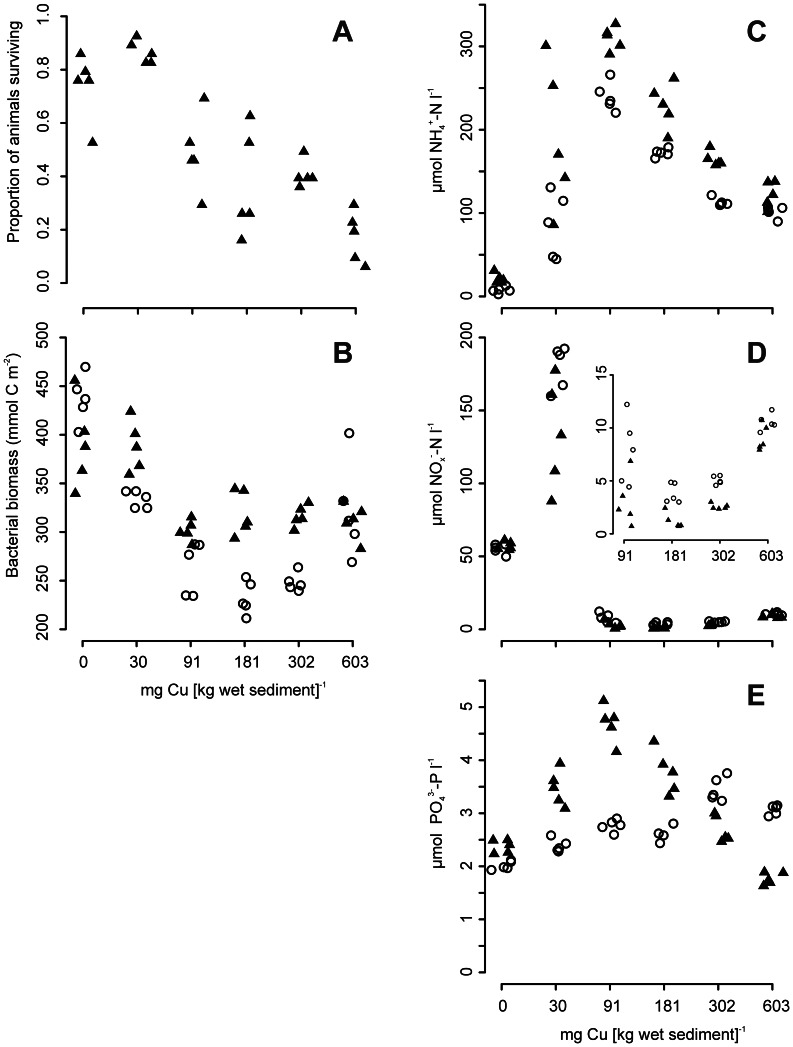
The effects of increasing copper concentration on (a) the proportion of *C. volutator* surviving, (b) bacterial biomass, and concentrations of (c) NH_4_^+^-N, (d) NO_x_^–^N and (e) PO_4_^3–^P. Mesocosms containing *C. volutator* are represented by filled triangles; those without are represented by open circles. The inset figure (d) is re-scaled for clarity. Data on the x-axis are jittered to facilitate data visualisation.

Copper concentration had a significant, negative effect on the survival of *C. volutator* (F = 22.01, df_5,24_, p<0.001; Figure 1a; Table 1), although differences between copper concentrations of 0 (control) and 30.2 mg Cu [kg wet sediment]^−1^ were not significant (Table S3). The estimated LC_50_ was 190 mg Cu [kg wet sediment]^−1^ with upper and lower 95% confidence intervals of 161 and 224 mg Cu [kg wet sediment]^−1^ respectively. This is in excellent agreement with our previous estimate of 193 (95% confidence intervals: upper = 171, lower = 219) mg Cu [kg wet sediment]^−1^ [19], demonstrating that the concentrations of copper in the experimental sediments and overlying waters were effectively the same as measured previously (Table S1).

### Copper and *C. volutator* Effects on Nutrient Concentrations and Bacterial Biomass and Sediment δ^13^C

It was necessary to account for the different levels of variance (heteroscedasticity) observed across the copper treatments by including copper concentration as a variance covariate in all of the analyses (p≤0.027 in all cases; Table 2). Bacterial biomass and all nutrient concentrations at the end of the 10-day experiment were affected by significant copper × *C. volutator* interactions (p≤0.011 in all cases; Table 2; Figure 1b–e). Bacterial biomass clearly declined in response to the copper additions, but remained higher in the presence of *C. volutator* at concentrations between 30 and 302 mg Cu [kg wet sediment]^−1^ (Figure 1b). Concentrations of NH_4_^+^-N in all copper-spiked mesocosms were above those in the controls. They reached a maximum at 91 mg Cu [kg wet sed]^−1^ (Figure 1c) and declined thereafter. The net release of NH_4_^+^-N after 10 days was greater in the presence of *C. volutator* at all but the highest concentration of copper. All levels of copper contamination, excluding the lowest treatment, resulted in concentrations of NO_x_^–^N being lower than those in the controls (Figure 1d). The presence of *C. volutator* resulted in lower mean concentrations of NO_x_^–^N in all copper treatments, with the relative difference being greatest at 302 mg Cu [kg wet sed] ^−1^.

**Table 2:** Summary of statistical models investigating the factors influencing nutrient concentrations and bacterial biomass.

Response	Model term	df	L. ratio	*p*
NH_4_^+^-N	Copper × *C. volutator*	5	45.42	<0.001
	Copper^1^	5	70.08	<0.001
NO_x_^–^N	Copper × *C. volutator*	5	14.82	0.011
	Copper^1^	5	166.84	<0.001
PO_4_^3–^P	Copper × *C. volutator*	5	111.73	<0.001
	Copper^1^	5	15.14	0.010
Bacterial biomass	Copper × *C. volutator*	5	30.49	<0.001
	Copper^1^	5	15.75	0.008

1 Variance covariate.

In the absence of *C. volutator*, there was a positive association between copper concentrations and the net accumulation of PO_4_^3–^P in the overlying water (Figure 1e). When *C. volutator* was present, concentrations of PO_4_^3–^P also increased across the control and two lowest concentrations of copper and then declined rapidly as copper contamination increased further.

### Copper and *C. volutator* Effects on Microbial PLFAs

**Figure 2:**
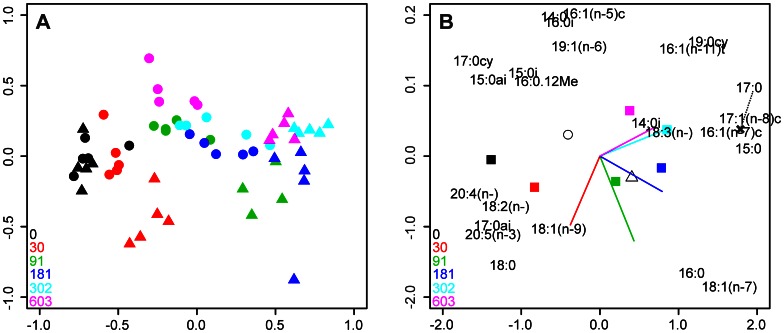
Redundancy analysis (RDA) distance triplot of the mol % PLFA data visualising (a) the 60 independent mesocosm experiments (‘sites’) and (b) the 24 PLFAs (‘species’) and the effects of the explanatory variables. Colours relate to the nominal sediment concentration of copper, as denoted by the inset legend (units = mg Cu [kg wet weight]^−1^). Filled triangles and circles represent mesocosms with and without *C. volutator* present respectively. Explanatory variables used were nominal copper concentrations (filled squares), the presence/absence of *C. volutator* (open triangle and circle respectively) and a copper × *C. volutator* interaction (plotted as vectors by copper concentration). The original location of the fatty acid 17∶0 is indicated by a black cross symbol. A single replicate (green triangle; 91 mg Cu [kg wet weight]^−1^ with *C. volutator*; x = 0.034, y = −1.754) was omitted to facilitate data visualization. Double bond positions for the PLFAs 18∶2(n−6,9), 18∶3(n−5,10,12) and 20∶4(n−5,8,11,14) were omitted for clarity. Parts (a) and (b) are plotted separately to facilitate data visualisation.

Absolute concentrations of individual PLFAs, their relative composition (mol %) and isotopic signatures (δ^13^C) are presented in Figures S1, S2 and S3 respectively. In order of importance, copper concentration, the presence of *C. volutator* and an interaction between these variables all significantly (p<0.001) increased the amount of variance explained in the percentage (mol %) PLFA data (Table S4). The amount of variation purely attributable to copper and *C. volutator* was 42% and 12% respectively. A total of 67% of the variation in the data was explained by all the explanatory variables and 49% of all variation was explained by the first two axes (Table S5). The resulting RDA triplot (Figure 2) visualises the additive and interactive effects of copper and *C. volutator* on the relative abundance of PLFAs in the sediments. The PLFA signature in the control treatments (black symbols) was distinct from all others and did not differ due to the addition of *C. volutator*. Increasing copper concentration resulted in a progressive shift in the composition of PLFAs (imagine a vertical line of origin x = −0.75, y = 0.5 rotating anticlockwise as copper concentration increases); the relative change in the PLFA signature decreased as copper concentration increased. For a given concentration of copper, the addition of *C. volutator* resulted in a distinct shift in the relative abundance of the PLFAs (compare filled circles and triangles for any given colour but black).The control and lowest copper treatment were largely discriminated on the first axis, which had strong negative loadings of the PLFAs 20∶4(n–5, 8, 11, 14), 17∶0cy, 15∶0ai, 20∶5(n–3) and 17∶0ai. Mesocosms containing *C. volutator* at copper concentrations ≥91 mg Cu [kg wet weight]^−1^ were also mainly discriminated on the first axis, with strong positive loadings of 15∶0, 17∶1(n–8), 17∶0, 18∶1(n–7) and 16∶1(n–7).

**Figure 3:**
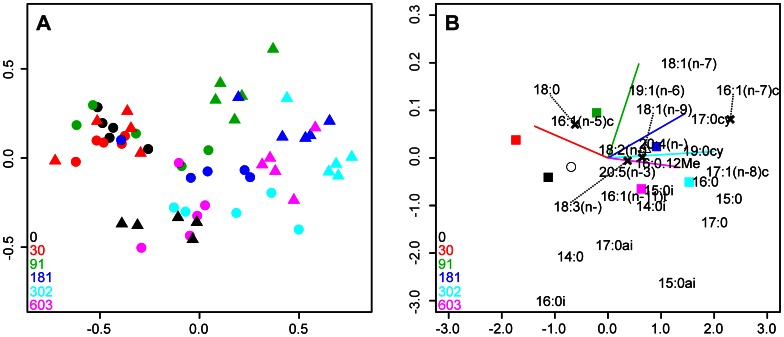
Redundancy analysis (RDA) distance triplot of the δ^13^C PLFA data visualising (a) the 60 independent mesocosm experiments (‘sites’) and (b) the 24 PLFAs (‘species’) and the effects of the explanatory variables. Colours relate to the nominal sediment concentration of copper, as denoted by the inset legend (units = mg Cu [kg wet weight]^−1^). Filled triangles and circles represent mesocosms with and without *C. volutator* present respectively. Explanatory variables used were nominal copper concentrations (filled squares), the presence/absence of *C. volutator* (open triangle and circle respectively) and a copper × *C. volutator* interaction (plotted as vectors by copper concentration). The original locations of the fatty acids 16∶1(n−7)c, 18∶0, 18∶1(n−1) and 18∶3(n−5,10,12) are indicated by black cross symbols. Double bond positions for the PLFAs 18∶2(n−6,9), 18∶3(n−5,10,12) and 20∶4(n−5,8,11,14) were omitted for clarity. Parts (a) and (b) are plotted separately to facilitate data visualisation.

The amount of explained variance in the δ^13^C PLFA data increased significantly (p<0.001) by incorporating *C. volutator*, nominal copper concentrations and their interaction (Table S4). Copper and *C. volutator* individually explained 19% and 7% of the variance in the data. The total amount of variance explained by the explanatory variables was 45%; 29% of the variance in the δ^13^C PLFA data was explained by the first two axes (Table S5). The RDA triplot (Figure 3) revealed similar albeit it less prominent patterns to those in Figure 2. The δ^13^C values of the sediment PLFAs changed in response to increasing concentrations of copper. The presence of *C. volutator* also resulted in a pronounced change in the isotopic signature of the PLFA suite; the relative magnitude of the shift was dependent upon copper concentration. The control treatment where *C. volutator* was excluded and all mesocosms spiked with copper at 30 mg Cu [kg wet weight]^−1^ discriminated on axis 1, with negative loadings of the PLFAs 16∶0i, 14∶0, 16∶1(n−5) and 18∶0. Mesocosms containing *C. volutator* and copper at concentrations ≥91 mg Cu [kg wet weight]^−1^ also discriminated predominantly on axis 1, with positive loadings of 17∶1(n−8)c, 15∶0, 16∶1(n−7), 17∶0, 17∶0cy, 16∶0 and 19∶0cy.

## Discussion

Our study demonstrates that the effects of copper contamination on the structure and metabolic functioning of a soft sediment benthic microbial community are different in the presence of macrofauna. Copper × *C. volutator* interactions affected bacterial biomass, nutrient concentrations, microbial community structure and their isotopic signatures at the end of our experiment.

### Copper and Macrofaunal Effects on Sediment Nutrient Exchange

*C. volutator* are known to stimulate biogeochemical cycling in marine sediments through their ventilatory activities [31], [32], and can up-regulate their metabolic- and hence excretion rates in response to heavy metal contamination [31], [51], [52]. Elevated levels of NH_4_^+^-N and PO_4_^3–^P in the presence of *C. volutator* were therefore expected. However, our experimental sediments also contained bacteria and microalgae, both of which also contribute significantly to benthic elemental cycling.

Copper has direct, adverse affects on bacteria [16], [3] and microalgae [53], [54]. *C. volutator* affects these two groups of organisms directly through their feeding [55], [56], [57]. They also affect them indirectly via bioturbation [31], [32] and their capacity to bioaccumulate copper and hence detoxify the surrounding environment [58], [59]. In addition, the movement and burrowing activity of *C. volutator* changes in response to increasing copper concentrations [19], [59], further affecting the supply and distribution of oxygenated seawater and bioavailable copper to benthic organisms [8]. We suggest that the typically lower concentrations of NOx^–^N observed in the mesocosms containing *C. volutator* indicates that the presence and activity of these animals provided some, albeit variable, alleviation of copper-inhibited denitrification for the resident microbial community [29], [31]. This interpretation is consistent with the understanding that denitrification is the dominant loss process for nitrate in intertidal sediment ecosystems [60], [61], [62] and the known sensitivity of denitrifying bacteria to copper [29], [63].

Looking beyond the interactive effects of copper and *C. volutator*, broad similarities exist in the patterns of net nutrient fluxes across the different levels of copper either with or without *C. volutator*. This observation is consistent with the understanding that the direct effects of copper on microbe-mediated nutrient cycling are greater than indirect effects caused by impaired- or lost macrofaunal functionality and species identity [20]. Concentrations of NH_4_^+^-N and NO_x_^–^N were elevated and depressed respectively relative to the controls at all but the lowest copper treatment (Figures 1d & e). The high concentrations of NO_x_^–^N observed in the lowest copper treatment likely reflect the stimulatory effect that low concentrations of this metal have on nitrification [63], [64]. Lower concentrations of NO_x_^–^N at levels of copper beyond this do not reflect increased uptake by benthic microalgae as NH_4_^+^-N concentrations, which phytoplankton preferentially utilise over NO_3_^−^ [65], remained above control values at all levels of copper contamination. It is also unlikely that this effect reflects an increase in denitrification as this process is negatively affected by copper [29], [63]. Rather, the positive effect of copper on the net accumulation of NH_4_^+^-N indicates an increase in heterotrophy of the benthic community [66] and reduced nutrient uptake by benthic microalgae. The reduced accumulation of NO_x_^–^N was likely due to copper inhibition of nitrification [31]. The negative relationship between copper concentration and the algal biomarker polyunsaturated fatty acids (PUFAs), 18∶2(n−6,9), 20∶4(n−5,8,11,14) and 20∶5(n−3) (Figures S1 andS2) demonstrates that copper contamination had a strong, negative effect on the microphytobenthos in our mesocosms [53]. This observation is consistent with the aforementioned mechanism to explain the observed accumulation of NH_4_^+^-N in the overlying waters. Nevertheless, further work across a variety of scales is required to confirm the mechanisms suggested above. Highly targeted laboratory experiments using mono- and multi-species mixtures of benthic microbes and macrofauna are necessary to provide mechanistic insights into the interrelationships between copper contamination and the ways in which organisms interact with elemental cycles. Equally, field-scale observations across a variety of locations will be necessary to ‘ground-truth’ the existence and relevance of such effects in the natural world.

### Copper and Macrofaunal Effects on the Sediment Microbial Community

Copper has been shown to increase maintenance energy demands in estuarine microbial communities, requiring an increasing proportion of the available substrates to be channelled towards catabolic processes as contamination levels increase [3], [66], [67]. The reduction in bacterial biomass with increasing copper concentrations was therefore expected. However, the typically positive effect of *C. volutator* on bacterial biomass is in contrast to the reported negative effects of this animal on microbial biomass in estuarine sediments [55]. We attribute this discrepancy to the increased availability of metabolic substrates for bacterial growth in the form of dead *C. volutator* in our experiments (discussed below).

The significant copper × *C. volutator* interactions observed in the PLFA relative abundance- (Figure 2) and isotopic data (Figure 3) demonstrate that the effects of copper on the structure and metabolic functioning of the sediment microbial community differ in the presence of *C. volutator*. This result is consistent with the known effects of bioturbating organisms on microbial community structure [22], [23] and the capacity for *C. volutator* to influence copper bioavailability [58], [59]. Considering the effects of copper on bacteria and microphytobenthos discussed above, it is not surprising that sediments from the control- and lowest copper treatments were discriminated from others by generic PLFA biomarkers for bacteria (17∶0cy, 15∶0ai and 17∶0ai) and diatoms (20∶4(n−5, 8, 11, 14), 20∶5(n−3)) [68]; the highest quantities of these two groups of organisms were present in the control cores.

Discerning the mechanisms causing the observed changes in the isotopic signatures of individual PLFAs is somewhat more complex. Carbon isotope signatures reflect a variety of factors, including the signatures of basal resources and specific metabolic pathways that result in isotopic fractionation [34], [36]. Indeed, recent work in an undisturbed soil ecosystem has highlighted the paucity of knowledge on the turnover rates of individual groups of microorganisms and the isotopic fractionations that result from their specific metabolic pathways [69]. Even less is known about these issues in natural marine microbial communities. Potential modifications in sediment oxygen concentrations, driven by the interactive effects of bioturbation [31], [32] and copper [70], could have influenced the δ^13^C signatures of PLFAs both directly and indirectly. Discrimination during bacterial lipid biosynthesis can depend upon respiratory conditions [71]. Any change in the relative abundance of the bacteria responsible for anaerobic ammonia oxidization, a process that is widespread in soft sediment habitats [72], will influence the observed changes in δ^13^C of individual PLFAs. Pure culture experiments with these organisms demonstrate that they strongly fractionate against ^13^C [73]. However, the first principle component in the redundancy analysis of δ^13^C signatures of the PLFAs (Figure 3) had strong, positive loadings of the PLFAs 17∶1(n−8), 15∶0, 16∶1(n−7), 17∶0, 17∶0cy, 16∶0 and 19∶0cy. Many of these are typically associated with sulfate-reducing bacteria [74], [75], [76]. It therefore seems likely that the observed changes in δ^13^C were at least partially attributable to this group of organisms, which predominate in shallow water sediments [77], including those used in the present study [78]. Cultured sulfate-reducing bacteria typically produce ^13^C depleted PLFAs, with the extent of isotopic discrimination depending upon whether they are undergoing auto-, mixo- or heterotrophic growth [76]. This explanation is incomplete, however, as the generic bacterial PLFAs 15∶0, 15∶0ai and 17∶1(n−8) became progressively ^13^C enriched as copper concentrations increased (Figure S3). We suggest that this phenomenon can be explained by the increased bacterial utilisation of dead *C. volutator* biomass as copper concentrations increased. The δ^13^C signature of *C. volutator* collected from the same location as our experimental animals, approximately −15.8 ‰ [79], is considerably greater than the value of −22.3 ‰ observed for the bulk sedimentary organic material. There is a need for a more detailed understanding of the processes that influence the isotopic signatures of organisms, particularly as the application of compound-specific techniques such as those used herein are likely to become more commonplace in the future.

### Copper and Macrofaunal Effects in Natural Sediment Ecosystems

Our data were derived from a 10-day experiment conducted on defaunated sediments retrieved from an intertidal mudflat. We made no attempt to allow the microbial community to adapt or shift towards copper tolerance, although this process was almost certainly taking place over the experimental duration [63]. We also added only a single invertebrate species which is clearly an over simplification of the natural world. Such limitations are typical of mesocosm-type experiments, and must be carefully considered when attempting to place the resulting data into a wider ecological and biogeochemical context [80]. It is conceivable that the strong, interactive effects between copper concentration and the presence of a bioturbating organism reported herein are only applicable to our experimental system. However, we suggest that our findings are more widely applicable because a) our original hypothesis was developed from the understanding that copper and bioturbation both affect the structure and functioning of microbial communities across a range of habitats and ecosystems [18], [21], [22], [30], [32]; b) the reported effects of copper and *C. volutator* are consistent with previous research conducted over different timescales, in different locations using different organisms and techniques. We therefore contend that the assimilative capacity of any marine soft sediment benthic community will become progressively impaired by any process that causes the concentrations and bioavailability of copper to increase. Reductions of benthic bacterial- and microalgal biomass will decrease the capacity of these organisms to process elements such as carbon and nitrogen. This is particularly important in the global context of aquaculture activities, which must double by 2050 if current per capita consumption rates are to be sustained [81]. Marine fish farming can result in the accumulation of both organic matter and copper in the underlying sediments [9], [13], [46]. It follows that copper contamination will serve as a positive feedback mechanism for organic enrichment in such environments. Decreased availability of benthic bacteria and microalgae will also negatively impact upon the energetic and nutritional value of contaminated sediments, particularly as PUFAs such as 20∶5(n−3) are widely considered to be essential for many marine organisms.

Our data demonstrate that the effects of copper contamination on the structure and functioning of soft sediment habitats cannot be predicted solely from ambient concentrations of this heavy metal. The macrofaunal community at any particular location will influence, and in certain cases alleviate, the negative effects of copper contamination on the assimilative capacity of the local environment. Other processes that influence the flushing of sediments with fresh, aerated seawater, such as storm surge events, may also be expected to have similar effects. These findings have serious implications for environmental managers and marine policy makers; they indicate that a concentration-based approach to environmental management will yield unsatisfactory results across multiple benthic habitats. Indeed, they support the move towards an ecosystem approach to environmental management that places increased emphasis on the biological and ecological characteristics of each given location [30], [82]. The successful and widespread application of the ecosystem approach will require increased efforts to investigate if and how pollutants influence the structure and functioning of marine communities across a range of environments and seasons.

## Supporting Information

Figure S1 **Concentrations (± SE) of individual PLFAs measured at the end of the experiment.**
(DOC)

Figure S2 **Relative concentrations (mol %, ± SE) of individual PLFAs measured at the end of the experiment.**
(DOC)

Figure S3 **Isotopic composition (δ^13^C, ± SE) of individual PLFAs measured at the end of the experiment.**
(DOC)

Table S1 **Nominal and measured concentrations of copper in the sediments and overlying waters of a previous, identical experiment with** ***C. volutator*** **after 10 days of incubation.**
(DOC)

Table S2 **Percent of variation in nutrient concentrations at the end of the experiments attributable solely to copper concentration and square root arcsin transformed proportion of** ***C. volutator*** **surviving.**
(DOC)

Table S3 **Model output from the analysis examining how copper dose (0–6) affected the square root arcsin transformed proportion of** ***C. volutator*** **surviving the 10-day incubation period.**
(DOC)

Table S4 **Order of importance, F-statistics, p-values and the % variance solely attributable to each variable in the two RDA analyses.**
(DOC)

Table S5 **Numerical output from RDA analysis of the mol % and δ^13^C PLFA data.**
(DOC)

Text S1 **Estimated metabolic demands of** ***C. volutator*** **over the 10-day experimental duration.**
(DOC)
