# Supplementary material for: Metal-Macrofauna Interactions Determine Microbial Community Structure and Function in Copper Contaminated Sediments
Source: PLoS One. 2013 May 31;8(5):e64940. doi: 10.1371/journal.pone.0064940 (PMC3669130; doi:10.1371/journal.pone.0064940)

**Figure S2.** Relative concentrations (mol %, ± SE) of individual PLFAs measured at the end of the experiment. Filled triangles and open circles represent mesocosms with and without *C. volutator* present respectively.


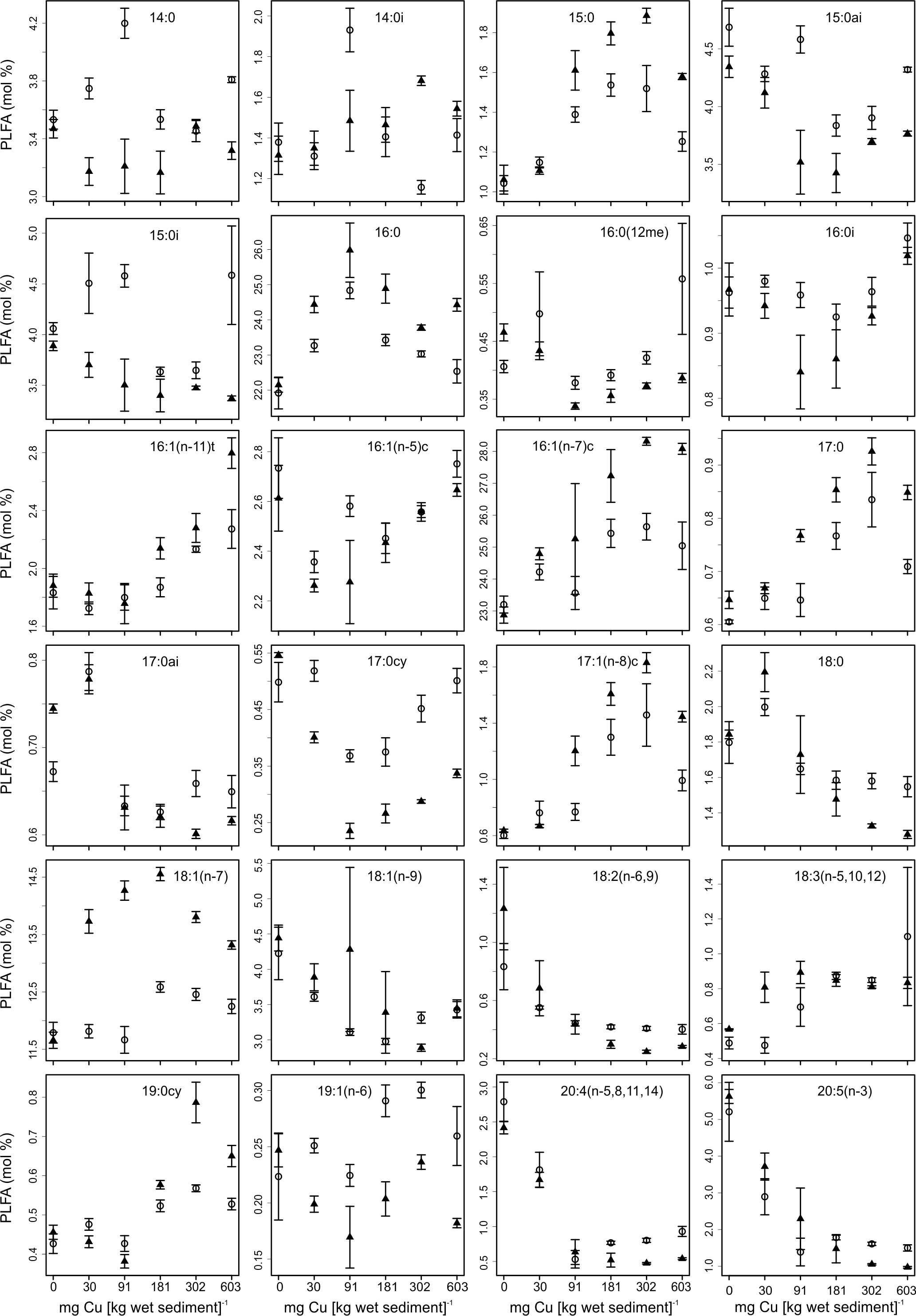

Supplement: Figure S2 — Relative concentrations (mol %, ± SE) of individual PLFAs measured at the end of the experiment. (DOC) [file pone.0064940.s002.doc]
