# Supplementary material for: Metal-Macrofauna Interactions Determine Microbial Community Structure and Function in Copper Contaminated Sediments
Source: PLoS One. 2013 May 31;8(5):e64940. doi: 10.1371/journal.pone.0064940 (PMC3669130; doi:10.1371/journal.pone.0064940)
Supplement: Table S1 — Nominal and measured concentrations of copper in the sediments and overlying waters of a previous, identical experiment with C. volutator after 10 days of incubation. (DOC) [file pone.0064940.s004.doc]

**Table S1.** Nominal and measured concentrations of copper in the sediments and overlying waters of a previous, identical experiment with *C. volutator* after 10 days of incubation. ND = not determined. Concentrations of copper in the sediments and solution were determined using X-ray fluorescence (Niton XLT, UK) and furnace atomic absorption spectrometry (Perkin Elmer AAnalyst 100; Perkin Elmer, USA) respectively.

| Nominal | Measured | |
| --- | --- | --- |
| Sediment [Cu]  (mg Cu [kg wet sediment]^-1^) | Sediment [Cu]  (mg Cu [kg wet sediment]^-1^) | Dissolved [Cu]  (mg Cu Liter^-1^) |
| 0.0 | ND | 0.064 ± 0.011 |
| 30.2 | 42.8 ± 3.3 | 0.108 ± 0.037 |
| 90.5 | 91.9 ± 28.3 | 0.132 ± 0.016 |
| 181.0 | 136.8 ± 45.7 | 0.208 ± 0.035 |
| 301.7 | 262.7 ± 26.0 | 0.280 ± 0.039 |
| 603.4 | 366.4 ± 29.0 | 0.860 ± 0.073 |
