# Supplementary material for: Metal-Macrofauna Interactions Determine Microbial Community Structure and Function in Copper Contaminated Sediments
Source: PLoS One. 2013 May 31;8(5):e64940. doi: 10.1371/journal.pone.0064940 (PMC3669130; doi:10.1371/journal.pone.0064940)
Supplement: Table S2 — Percent of variation in nutrient concentrations at the end of the experiments attributable solely to copper concentration and square root arcsin transformed proportion of C. volutator surviving. (DOC) [file pone.0064940.s005.doc]

**Table S2.** Percent of variation in nutrient concentrations at the end of the experiments attributable solely to copper concentration and square root arcsin transformed proportion of *C. volutator* surviving. Shared = total amount of shared variance.

|  | NH_4_^+^-N | NO_x_^-^-N | PO_4_^3-^-P |
| --- | --- | --- | --- |
| Copper | 86.1 | 38.4 | 87.6 |
| Survival | 0.2 | <0.1 | 1.1 |
| Shared | 1.1 | 54.4 | 5.8 |
