# Supplementary material for: Metal-Macrofauna Interactions Determine Microbial Community Structure and Function in Copper Contaminated Sediments
Source: PLoS One. 2013 May 31;8(5):e64940. doi: 10.1371/journal.pone.0064940 (PMC3669130; doi:10.1371/journal.pone.0064940)
Supplement: Table S3 — Model output from the analysis examining how copper dose (0–6) affected the square root arcsin transformed proportion of C. volutator surviving the 10-day incubation period. (DOC) [file pone.0064940.s006.doc]

**Table S3.** Model output from the analysis examining how copper dose (0-6) affected the square root arcsin transformed proportion of *C. volutator* surviving the 10-day incubation period. All comparisons relate to the model baseline, which is set to copper dose 0 (control).

| Coefficient | Estimate | SE | t | p |
| --- | --- | --- | --- | --- |
| Intercept | 1.051 | 0.061 | 17.315 | < 0.001 |
| Dose 1 | 0.160 | 0.086 | 1.863 | 0.075 |
| Dose 2 | -0.273 | 0.086 | -3.174 | 0.004 |
| Dose 3 | -0.402 | 0.086 | -4.685 | < 0.001 |
| Dose 4 | -0.353 | 0.086 | -4.114 | < 0.001 |
