# Supplementary material for: Metal-Macrofauna Interactions Determine Microbial Community Structure and Function in Copper Contaminated Sediments
Source: PLoS One. 2013 May 31;8(5):e64940. doi: 10.1371/journal.pone.0064940 (PMC3669130; doi:10.1371/journal.pone.0064940)
Supplement: Table S4 — Order of importance, F-statistics, p-values and the % variance solely attributable to each variable in the two RDA analyses. (DOC) [file pone.0064940.s007.doc]

**Table S4.** Order of importance, F-statistics, p-values and the % variance solely attributable to each variable in the two RDA analyses. It was not possible to distinguish the variance associated with the interaction term due to collinearity with the two main terms.

|  | Order | Variable | F-statistic | p-value | Variance (%) |
| --- | --- | --- | --- | --- | --- |
| Mol % | 1 | Copper | 8.920 | <0.001 | 42 |
|  | 2 | *C. volutator* | 14.601 | <0.001 | 12 |
|  | 3 | *C. volutator* × Copper | 2.825 | <0.001 | NA |
|  |  |  |  |  |  |
| δ^13^C | 1 | *C. volutator* | 4.956 | <0.001 | 7 |
|  | 2 | Copper | 4.026 | <0.001 | 19 |
|  | 3 | *C. volutator* × Copper | 2.039 | <0.001 | NA |
