# Supplementary material for: Metal-Macrofauna Interactions Determine Microbial Community Structure and Function in Copper Contaminated Sediments
Source: PLoS One. 2013 May 31;8(5):e64940. doi: 10.1371/journal.pone.0064940 (PMC3669130; doi:10.1371/journal.pone.0064940)
Supplement: Table S5 — Numerical output from RDA analysis of the mol % and δ13C PLFA data. (DOC) [file pone.0064940.s008.doc]

**Table S5.** Numerical output from RDA analysis of the mol % and δ^13^C PLFA data. The total variation in all cases is 1.

|  | Mol % | |  | δ^13^C | |
| --- | --- | --- | --- | --- | --- |
| Axis | 1 | 2 |  | 1 | 2 |
| Eigenvalue | 0.36 | 0.12 |  | 0.22 | 0.08 |
| Eigenvalue as % of total variation | 36 | 12 |  | 22 | 8 |
| Eigenvalue as cumulative % of total variation | 36 | 49 |  | 22 | 29 |
| Eigenvalue as % sum of all canonical eigenvalues | 55 | 18 |  | 48 | 17 |
| Eigenvalue as cumulative % sum of all canonical eigenvalues | 55 | 73 |  | 48 | 65 |
| Total sum of all canonical eigenvalues | 0.67 | |  | 0.45 | |
| PLFA variation explained by first 2 axes (%) | 49 | |  | 29 | |
