# Supplementary material for: Metal-Macrofauna Interactions Determine Microbial Community Structure and Function in Copper Contaminated Sediments
Source: PLoS One. 2013 May 31;8(5):e64940. doi: 10.1371/journal.pone.0064940 (PMC3669130; doi:10.1371/journal.pone.0064940)
Supplement: Text S1 — Estimated metabolic demands of C. volutator over the 10-day experimental duration. (DOC) [file pone.0064940.s009.doc]

**Text S1.** Estimated metabolic demands of *C. volutator* over the 10-day experimental duration.

*Corophium* of 4mm = 0.231 mg dry weight (estimated from Boates & Smith, 1979)

Mean O_2_ consumption = 1.536 µl O_2_ [mg dry weight]^-1^ hr^-1^ @ 10˚C (estimated from Mclusky, 1969). Note that this value is considerably higher than the 0.7 µl O_2_ hr^-1^ reported by Møller & Riisgård (2006).

= 36.88 µl O_2_ [mg dry weight]^-1^ day^-1^ @ 10˚C

Total O_2_ consumption = 255.58 µl O_2_ core^-1^ day^-1^ @ 10˚C (0.23 *36.88*30 animals core^-1^)

= 11.4 µmol O_2_ core^-1^ day^-1^ @ 10˚C (255.58/22.4)

= 0.0114 mmol O_2_ core^-1^ day^-1^ @ 10˚C

= 0.0161 mmol O_2_ core^-1^ day^-1^ @ 15˚C assuming Q10 = 2

Sediment carbon = 1.4% dry weight

= 14 mg C [g dry weight]^-1^

= 1.167 mmol C [g dry weight]^-1^

= 116.7 mmol C in upper 2 cm of core

(1.167 * 100g of dry sediment in upper 2 cm of core)

Assuming that 1% of the carbon is sufficiently labile and available to *Corophium* and RQ = 1, there is sufficient carbon to sustain the 30 individuals for 72 days (116.7*0.01]/0.0161).

**References**

Boates JS, Smith PC (1979) Length-weight relationships, energy content and the effects of predation on *Corophium volutator* (Pallas) (Crustacea: Amphipoda). *Proceedings of the Nova Scotia Institute of Science*, **29**, 489-499.

Mclusky DS (1969) The oxygen consumption of *Corophium volutator* in relation to salinity. *Comparative Biochemistry and Physiology*, 29, 743-753.

Møller & Riisgård (2006) Filter feeding in the burrowing amphipod *Corophium volutator*. *Marine Ecology Progress Series*, **322**, 213-224.
